# Augmented *O*-GlcNAcylation alleviates inflammation-mediated colon carcinogenesis via suppression of acute inflammation

**DOI:** 10.3164/jcbn.17-106

**Published:** 2018-03-17

**Authors:** Yoshimasa Hirata, Takatoshi Nakagawa, Kazumasa Moriwaki, Eiko Koubayashi, Kazuki Kakimoto, Toshihisa Takeuchi, Takuya Inoue, Kazuhide Higuchi, Michio Asahi

**Affiliations:** 1 Department of Internal Medicine II, Faculty of Medicine, Osaka Medical College, 2-7 Daigaku-machi, Takatsuki, Osaka 569-8686, Japan; 2 Department of Pharmacology, Faculty of Medicine, Osaka Medical College, 2-7 Daigaku-machi, Takatsuki, Osaka 569-8686, Japan

**Keywords:** *O*-GlcNAcylation, *Ogt*-Tg, colon carcinogenesis, inflammation, NF-κB

## Abstract

Colon cancer prevalence is high worldwide. *O*-GlcNAcylation has been associated with tumor growth in various tissues, including the colon; however, its link to carcinogenesis is not fully understood. We investigated the association of *O*-GlcNAcylation with colon carcinogenesis using a 1,2-dimethylhydrazine/dextran sodium sulfate-induced colon carcinogenesis model in wild type and *O*-GlcNAc transferase-transgenic (*Ogt*-Tg) mice. The incidence of colon cancer was significantly lower in *Ogt*-Tg than in wild type mice. The colonic length was not shortened in *Ogt*-Tg mice, and NF-κB p65 phosphorylation was strongly suppressed, indicating that reduction of inflammation might be related to the alleviation of colon carcinogenesis. Dextran sodium sulfate-induced acute colitis mice were used to evaluate the effect of *O*-GlcNAcylation on inflammation at the maximal inflammation period. In *Ogt*-Tg mice, NF-κB p65 phosphorylation and interleukin-1β mRNA expression were suppressed. Histochemical staining demonstrated shedding of colon epithelial cells in wild type mice a few days after dextran sodium sulfate treatment, whereas they remained essentially intact in *Ogt*-Tg mice. There were no significant differences on histochemical staining in the remaining epithelia between groups. These data suggest that *O*-GlcNAcylation could prevent colon carcinogenesis through reducing acute maximum inflammation, suggesting modulation of *O*-GlcNAcylation as a novel therapeutic option.

## Introduction

Colorectal cancer is the second leading cause of cancer-related deaths in men and women in Western countries,^(1)^ and its incidence is gradually increasing in Japan as well. The most common pathway of colorectal cancer development is thought to be the adenoma-carcinoma sequence, in which carcinoma develops from an adenomatous polyp.^(2)^ The current practice of removing adenomatous polyps of the colon and rectum is based on the belief that this will prevent colorectal cancer.^(3)^ However, recent reports have described that this practice might not effectively prevent secondary cancer development,^(4)^ leading to the proposal of an alternative pathway of *de novo* colon carcinogenesis, which involves an aggressive growth phenotype and rapid infiltration of neighboring tissue and lymph nodes.^(5–7)^

*O*-linked β-*N*-acetylglucosamine (*O*-GlcNAc) modification (*O*-GlcNAcylation) is an important post-translational modification that modulates the function of many nuclear and cytoplasmic proteins.^(8)^ Changes in *O*-GlcNAc levels on key proteins have been implicated in the etiology of type II diabetes and Alzheimer’s disease.^(9,10)^ The addition and removal of *O*-GlcNAc on target proteins is catalyzed by *O*-GlcNAc transferase (OGT) and *O*-GlcNAcase (OGA), respectively.^(11)^ *O*-GlcNAc-modified proteins play diverse roles in transcriptional regulation, the cell cycle, signaling, stress, and differentiation, as well as colon cancer development.^(11)^

OGT has been documented to regulate the transcriptional machinery by modifying the many transcription factors involved in cancer-related processes.^(12)^ Notably, *O*-GlcNAc levels are significantly elevated in various cancer types, including those of the breast,^(13)^ prostate,^(14)^ colon,^(15)^ lung,^(16)^ pancreas^(17)^ and chronic lymphocytic leukemia.^(18)^ Studies of *in vivo* xenografts of breast,^(19)^ prostate^(14)^ and pancreatic cancers^(17)^ have shown a critical role for *O*-GlcNAcylation in tumorigenesis or metastasis.

Many reports have demonstrated that nuclear factor-kappa B (NF-κB) signaling is directly and indirectly affected by *O*-GlcNAcylation. However, no clear pattern in NF-κB activity in the presence of increased *O*-GlcNAc levels has been reported. The *O*-GlcNAcylation of RelA/p65, a subunit of NF-κB, is critical for activation of NF-κB signaling in mesangial cells and T- and B-lymphocytes.^(20,21)^ NF-κB p65 is *O*-GlcNAcylated on residue Thr-352, which is required for transcriptional activity under hyperglycemic conditions.^(22)^ In contrast, several reports have suggested that elevated *O*-GlcNAcylation attenuates NF-κB signaling activation in rat aortic smooth muscle cells and primary cultured cardiomyocytes.^(23,24)^ However, the regulatory mechanism of NF-κB via protein *O*-GlcNAcylation remains elusive.

Inflammation is an established risk factor for colon cancer.^(25)^ Ulcerative colitis, a common form of inflammatory bowel disease (IBD), has been associated with an increased risk of colorectal cancer.^(26)^ Activation of NF-κB signaling was detected in mucosal IBD cells and colorectal carcinoma patients^(27,28)^ and has been suggested to play a key role in linking intestinal inflammation with colorectal cancer development.^(29)^ The pivotal role of NF-κB signaling in colorectal cancer development is also supported by the fact that erythromycin, a macrolide antibiotics, which has been reported to be anti-inflammatory and -oxidative agent in mammalian cells, has been successfully suppressed intestinal tumors in mice.^(30)^ However, few studies have addressed how overexpression of *O*-GlcNAcylation affects colon cancer development. A combinatorial administration of colon carcinogen, either azoxymethane or dimethyl hydrazine (DMH) and dextran sulfate sodium (DSS) in mice was well known colon inflammatory carcinogenesis model.^(31)^ Here, we addressed how overexpression of *O*-GlcNAcylation affects colon cancer development using this colon carcinogenesis model in *Ogt* transgenic (*Ogt*-Tg) mice.^(32)^

## Materials and Methods

### Antibodies

Antibodies against OGT and NF-κB p65 were purchased from Santa Cruz Biotechnology (Dallas, TX). Anti-*O*-GlcNAc antibody was obtained from Novus Biologicals (Littleton, CO). Anti-phospho-NF-κB p65 [NF-κB(p)] was obtained from Cell Signaling Technologies (Danvers, MA).

### Animals

To examine the role of OGT and *O*-GlcNAcylation on various diseases, we established a mouse *Ogt*-Tg line of the C57BL/6j background.^(32)^ C57BL/6j mice were obtained from Japan SLC Inc. (Shizuoka, Japan). The mice were maintained in specific pathogen-free conditions, fed standard chow (CLEA Japan, Inc., Tokyo, Japan), and kept in a comfortable condition at 20°C with appropriate humidity and a 12 h light/dark cycle at the Division of Research Animal Laboratory, Osaka Medical College throughout the experiments. All procedures associated with pain were performed under appropriate anesthesia.

### Mouse model of colon inflammatory carcinogenesis

The mouse model for colon inflammatory carcinogenesis was generated with DMH/DSS treatment as described previously.^(33,34)^ In brief, wild type (WT) and *Ogt*-Tg mice received DMH at 20 mg/kg body weight subcutaneously three times within a week, and then chronic colitis was induced by the administration of two cycles of DSS consisting of 2.2% DSS for 7 days followed by water for 14 days for each cycle. Twenty-eight days later, the mice were euthanized and the colons were excised. These specimens were opened longitudinally and fixed on a cork board in 10% formalin. After the measurement of colon lengths, the specimens were stained with 0.2% methylene blue and colonic tumors were counted under a stereomicroscope. The colon tissues were subjected to hematoxylin and eosin staining (H&E staining), and the mucosa was scraped and subjected to Western blot analysis and real-time polymerase chain reaction (PCR) as described below.

### Mouse model of acute colitis

The mouse model of acute colitis was established with DSS treatment.^(35)^ In brief, WT and *Ogt*-Tg mice received DSS (2.2%) for 7 days followed by water for 2 days, and then the mice were euthanized and the colonic tracts were excised. The colon tissues were subjected to H&E staining, and the mucosa was scraped and subjected to Western blot analysis and real-time PCR as described below.

### H&E staining

Tissue specimens from WT and *Ogt*-Tg mice were fixed in 4% paraformaldehyde in phosphate-buffered saline, dehydrated in a graded ethanol series, and embedded in paraffin. Serial sections 3 µm thick were then prepared, and analyzed using H&E staining. These pathological sections were observed using a Nikon ECLIPSE 80i microscope (Nikon, Tokyo, Japan).

### Sodium dodecyl sulfate-polyacrylamide gel electrophoresis (SDS-PAGE) and Western blot analysis

The protein of the colonic mucosa was extracted with Potter-Elvehjem Tissue Grinder (Model LR-41C, Yamato Scientific Co. Ltd., Tokyo, Japan) at the maximum speed. The homogenates were centrifuged at 15,000 rpm for 10 min and the supernatants were subjected to SDS-PAGE followed by Western blot analysis after quantitation of the protein concentration. The protein on the polyvinylidene fluoride membrane (EMD Millipore, Billerica, MA) was probed with antibodies of interest. The images were captured with Chemidoc (Bio-Rad, Hercules, CA) and quantitated using Quantity One software (Bio-Rad).

### Real-time PCR analysis

The RNA was extracted and prepared using RNAzol (Molecular Research Center, Inc., Cincinnati, OH) according to the manufacturer’s instructions. The RNA was reverse-transcribed and then subjected to real-time PCR analysis with a Dice thermal cycler (TP870, Takara Bio Inc., Shiga, Japan) using PrimeScript RT Reagent Kit and SYBR Premix Ex Taq II (Takara Bio Inc.), respectively. The primers used in the study are shown in Table 1.

## Results

### Reduction of inflammatory colon carcinogenesis in *Ogt*-Tg mice

To gain insight into the role of protein *O*-GlcNAcylation on inflammatory colon carcinogenesis in colon cancer, we examined the differences in colon carcinogenesis between *Ogt*-Tg and WT mice using a DSS/DMH-induced inflammatory colon carcinogenic model.

**Fig. 1:**
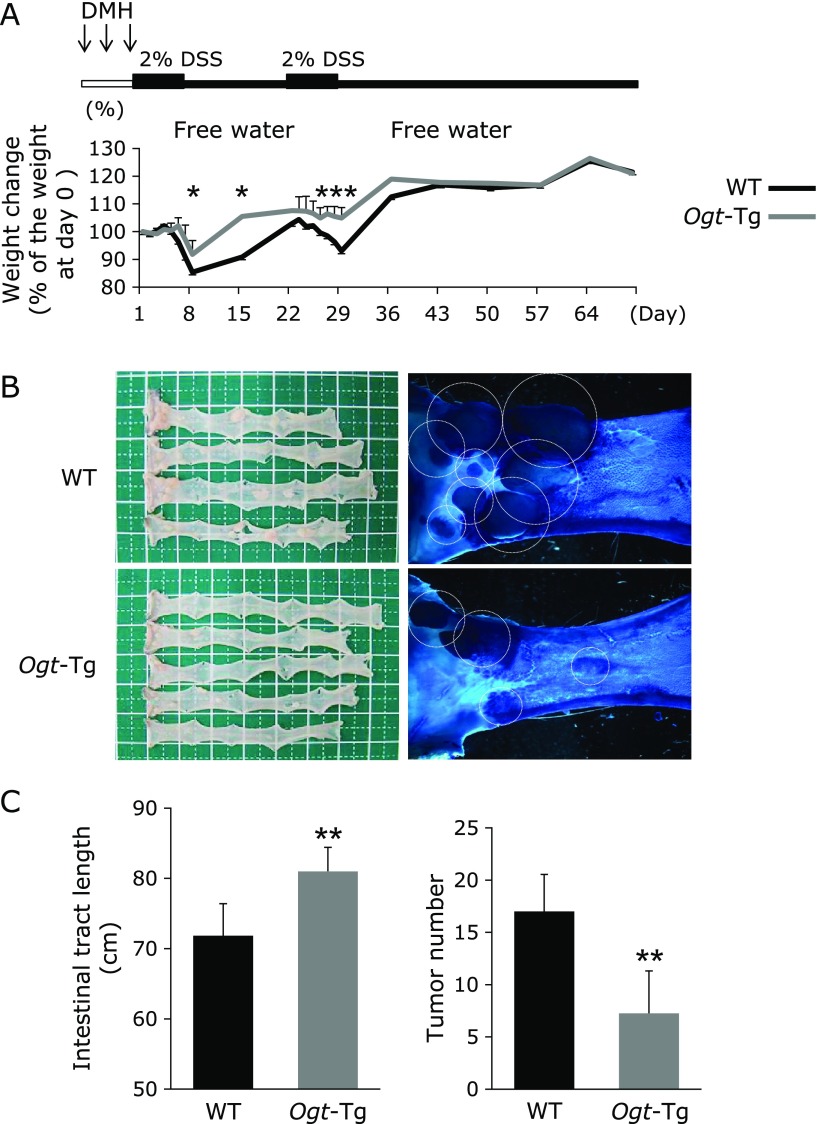
DSS/DMH induced colon carcinogenesis in WT and *Ogt*-Tg mice. DMH was administered subcutaneously three times (20 mg/kg) the 1st week, and then 2% DSS was supplied in the drinking water at the 2nd and 5th week. The mice were euthanized on day 70, and the colons were excised for analyses. (A) Schedule of the experiment and change of body weight during the experiment in WT and *Ogt*-Tg mice. *p<0.05. (B) Colons excised from WT and *Ogt*-Tg mice. Photograph showing methylene blue (MB) staining of the colons of the mice. The dotted circles indicate the MB-positive areas. (C) Comparison between WT and *Ogt*-Tg mice with respect to colon lengths and the number of tumors formed. **p<0.01.

**Fig. 2:**
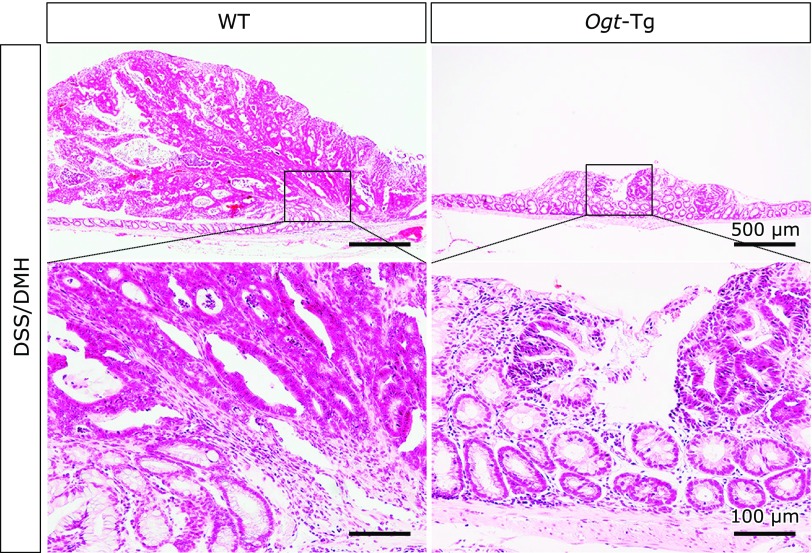
Hematoxylin and eosin (H&E) staining of colonic tissues in DMH/DSS-treated WT and *Ogt*-Tg mice. Tissue sections were stained with H&E and then analyzed as described in the Materials and Methods. Several sections were analyzed and representative images are presented.

The schedule of DMH administration (20 mg/kg, subcutaneous) and 2.2% DSS ingestion (2 periods, 1 week/period) is outlined in Fig. 1A. At day 8, 15, 27, 28 and 29 after the beginning of the first period of DSS ingestion, weight loss caused by DSS ingestion was significantly suppressed in *Ogt*-Tg mice compared to that observed in WT mice. To determine the number of tumors formed on colonic tissues, we stained the excised colons with 0.2% methylene blue. As shown in Fig. 1B and C, the number of tumors was also significantly lower in *Ogt*-Tg than in WT mice, and the tumors that grew in WT mice were much larger than those that grew in *Ogt*-Tg mice, indicating a higher probability of early tumor formation. The shortening of intestinal tract length was also significantly suppressed in *Ogt*-Tg mice than in WT mice (Fig. 1B and C). To confirm whether the tumors induced with DMH/DSS treatment were cancerous, we performed microscopic analysis of the mucosal epithelia of the mice irritated with DMH/DSS via H&E staining in WT and *Ogt*-Tg mice. The H&E staining of tumor sections in colonic tissues showed characteristic findings of cancer in all sections of the mucosal epithelia from both groups of mice (Fig. 2). Therefore, these results suggested that carcinogenesis could be alleviated in *Ogt*-Tg compared to WT mice.

**Fig. 3:**
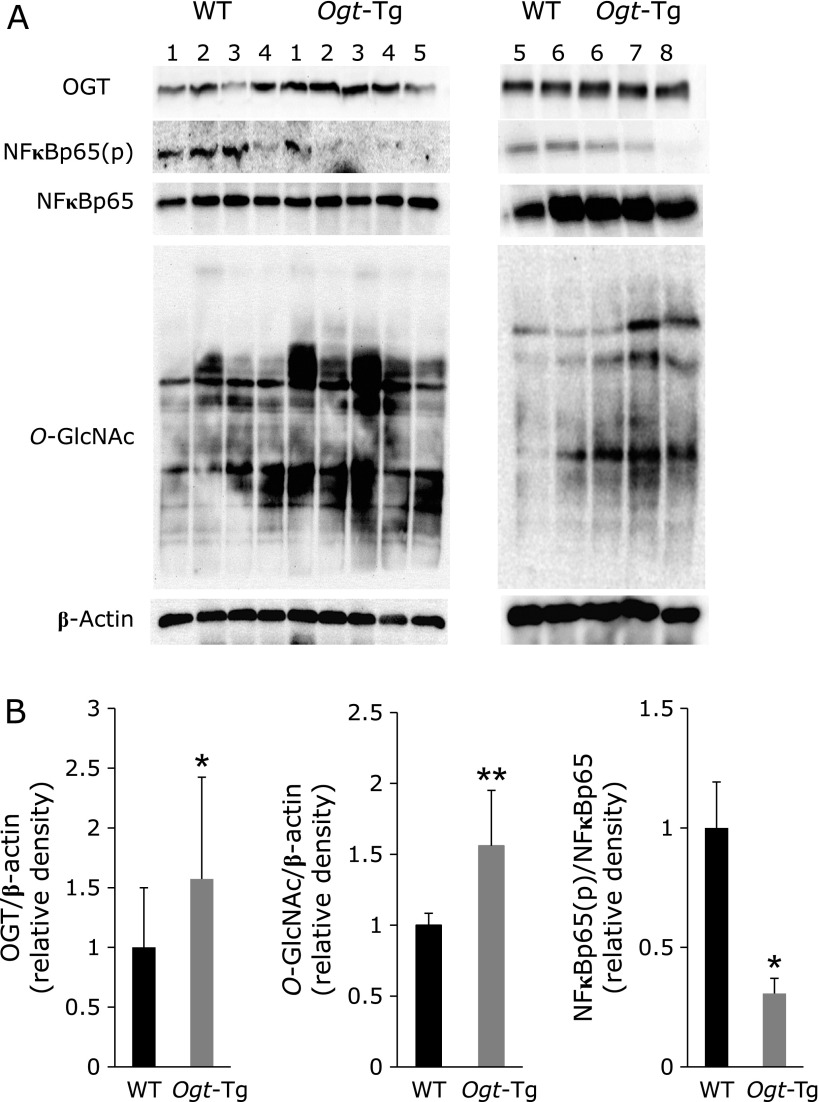
Western blot analysis of the colonic mucosa from DSS/DMH-treated WT and *Ogt*-Tg mice. (A) Western blot analysis. The experiment was performed several times and representative data from two runs are presented in the left and right panels. OGT, OGA, total and phospho-NF-κB [NF-κB(p)], *O*-GlcNAc, and β-actin levels were analyzed. (B) Densitometric analysis. The band densities in (A) were measured and analyzed. *p<0.05, **p<0.01.

Next, we performed Western blot analysis to investigate how carcinogenesis was alleviated in *Ogt*-Tg mice. As shown in Fig. 3A and B, we confirmed that the expression levels of OGT and the products, *O*-GlcNAcylated proteins, were significantly higher in *Ogt*-Tg than in WT mice. In addition, a dramatic change was observed in the level of NF-κB(p): the ratio of NF-κB(p) to total NF-κB was significantly lower in *Ogt*-Tg than in WT mice, suggesting that the inflammation induced by DSS/DMH treatment was considerably reduced in *Ogt*-Tg mice. Given that the colonic tissues were significantly longer in *Ogt*-Tg than in WT mice, OGT overexpression might reduce inflammation of the colon mucosa to protect the cells from inflammation-induced tumor formation. Surprisingly, the expression levels of proinflammatory cytokines such as *Il-1β*, *Il-6* and *Tnf-α* were not significantly different between the groups (data not shown).

### Inflammatory colitis in *Ogt*-Tg mice

**Fig. 4:**
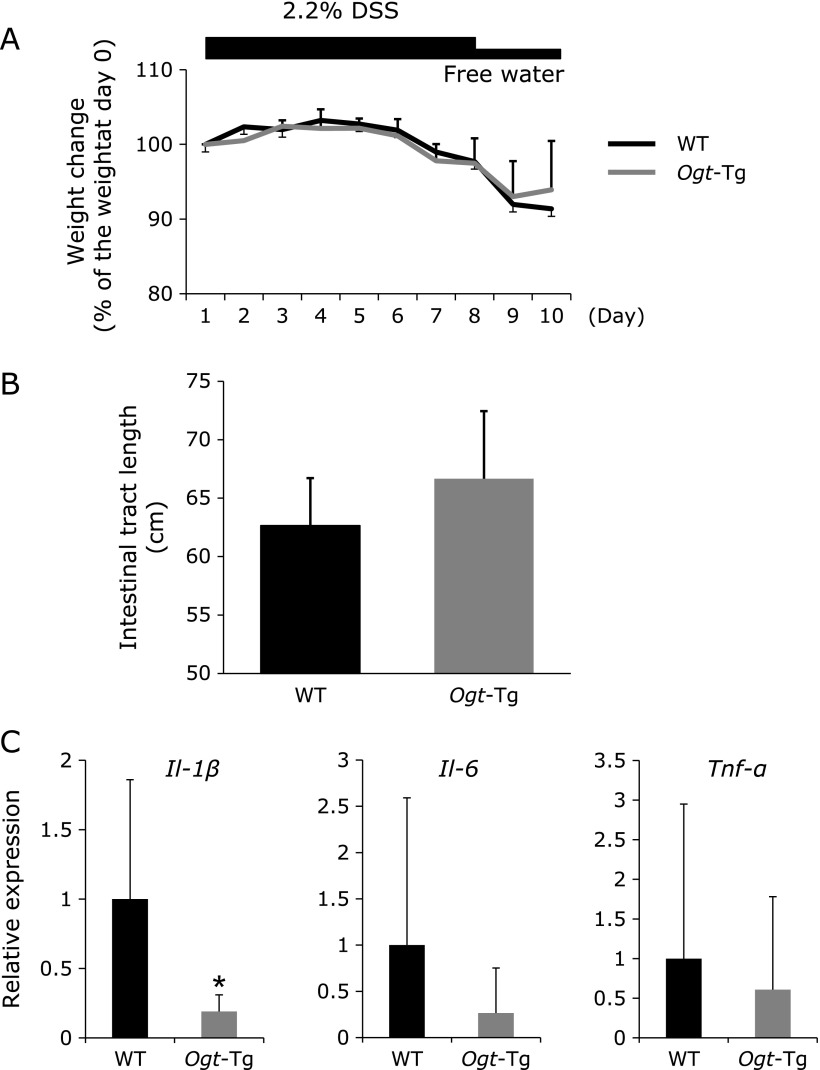
DSS-induced acute colitis in WT and *Ogt*-Tg mice. DSS (2.2%) was administered freely in the drinking water provided to WT and *Ogt*-Tg mice for 8 days, and the mice were put down and analyzed two days later. (A) Schedule of the experiment and averaged weight change of the mice. (B) Length of the intestinal tract of the mice. C. Real-time PCR analyses of cytokines (*Il-1β*, *Il-6* and *Tnf-α*) in the intestinal tract of mice treated with DSS. The values are expressed as the relative expression level, normalized to the level of *Gapdh*.

We hypothesized that *Ogt* overexpression prevented colon cancer formation induced by DMH/DSS via inhibition of inflammation, mainly the NF-κB pathway, resulting in discontinuation of inflammatory-induced carcinogenesis. To prove our hypothesis, we employed the DSS-induced acute colitis model, which involves only inflammation. Weight loss was milder than that in WT, which was apparent although not statistically significant (Fig. 4A). Shortening of the intestinal tract also tended to be alleviated in *Ogt*-Tg mice (Fig. 4B). To further confirm the compromised inflammation in *Ogt*-Tg compared to WT mice, real-time PCR analysis was performed, which revealed a significantly lower mRNA expression level of *Il-1β* in *Ogt*-Tg mice. The expression levels of other cytokines, including *Il-6* and *Tnf-α*, were also compromised in *Ogt*-Tg mice, although unfortunately these differences did not reach statistical significance (Fig. 4C), implying that *Ogt* overexpression could suppress acute inflammation.

**Fig. 5:**
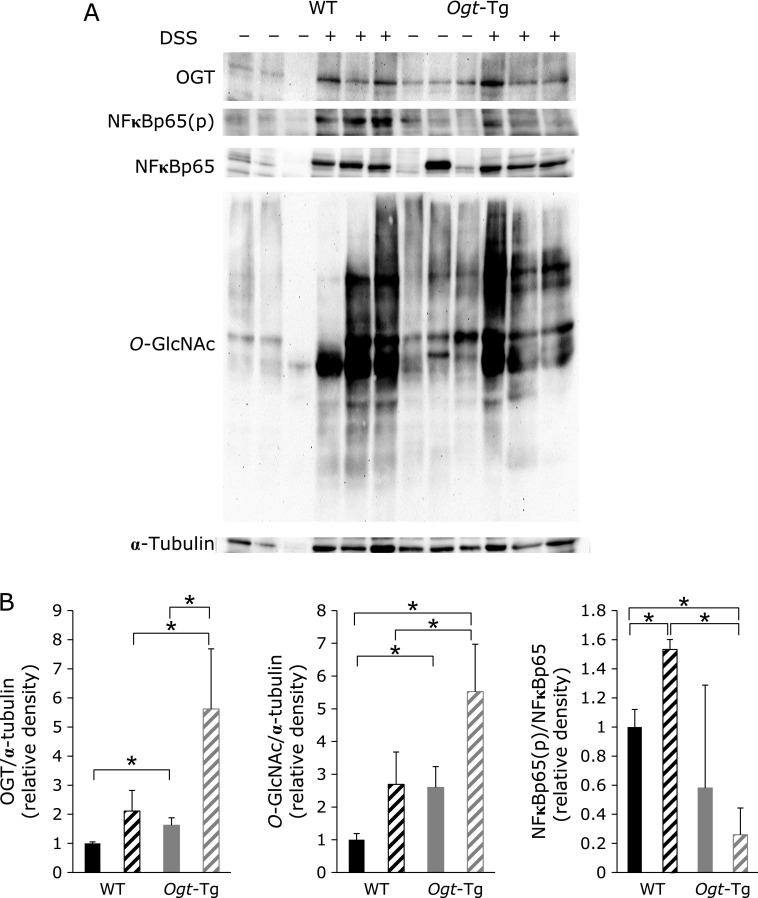
Western blot analysis of the colonic mucosa from DSS-treated WT and *Ogt*-Tg mice. (A) Western blot analysis. The experiment was performed several times and representative data are presented in the left and right panels, respectively. OGT, total and phospho-NF-κB [NF-κB(p)], *O*-GlcNAc, and α-tubulin were analyzed. (B) Densitometric analysis. The band densities in (A) were measured and analyzed. Black and gray represent WT and *Ogt*-Tg, respectively. Solid and hatched bars indicate DSS(–) and DSS(+), respectively. *p<0.05.

Western blot analysis was performed to further analyze the acute inflammation model in *Ogt*-Tg mice. Without administration of 2.2% DSS in the drinking water, OGT and *O*-GlcNAc expression levels were significantly higher in *Ogt*-Tg than in WT mice, which was not changed upon DSS administration. In contrast, no significant differences were observed in NF-κB and NF-κB(p) levels without DSS (Fig. 5A). However, upon DSS administration, the OGT level was seemingly elevated in both *Ogt*-Tg and WT mice. Densitometry analysis also revealed that OGT and *O*-GlcNAc levels were increased in both *Ogt*-Tg and WT mice, although the change of *O*-GlcNAc levels was not statistically significant (Fig. 5B). Both NF-κB(p) and NF-κB levels were also seemingly increased in WT mice, but were not substantially affected in *Ogt*-Tg mice (Fig. 5A). In contrast, the ratio of NF-κB(p) to NF-κB was significantly compromised in *Ogt*-Tg compared to WT mice (Fig. 5B). Taken together, these results demonstrate that DSS-induced acute inflammation was significantly suppressed in *Ogt*-Tg compared to WT mice, thereby confirming our hypothesis.

**Fig. 6:**
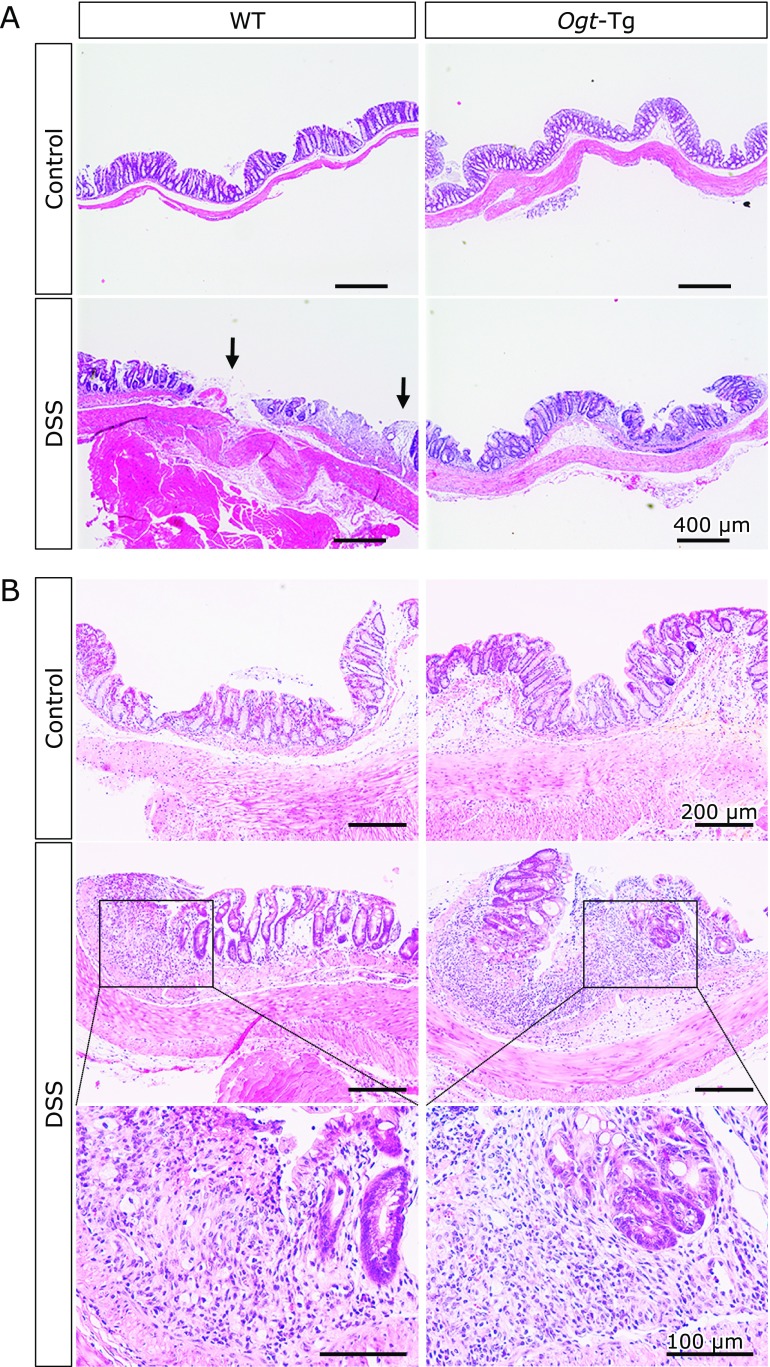
H&E staining of colon tissues in DSS-free [DSS (–)] (A) and DSS-treated [DSS (+)] (B) WT (left panel) and *Ogt*-Tg (right panel) mice. The inset indicates the location in the magnified image (10×). Scale bar: 200 µm.

Finally, we analyzed the colonic mucosal epithelia of the mice treated with DSS. The vertical section of the tissues was subjected to H&E staining as described in the Materials and Methods. We could not detect any significant differences between the H&E-stained tissues from WT and *Ogt*-Tg mice without DSS treatment, whereas many immune cells were found to have infiltrated the mucosal epithelia in DSS-treated mice, consistent with the weight loss patterns observed in the mice. Unfortunately, there was no obvious difference between WT and *Ogt*-Tg mice detected despite careful examination of the tissues. Nonetheless, considering the compromised inflammation implicated with the phosphorylation status of NF-κB and expression of *Il-1β*, *Il-6* and *Tnf-α* in *Ogt*-Tg mice (Fig. 6), we concluded that the higher OGT expression and subsequent protein *O*-GlcNAcylation in *Ogt*-Tg mice alleviated the DSS-induced acute inflammatory damages of the mucosal epithelia through the suppression of inflammatory cytokines and associated signaling pathways.

## Discussion

We revealed that *Ogt* overexpression compromised colon cancer formation in a DMH/DSS-induced inflammatory carcinogenic mice model. In *Ogt*-Tg mice, proinflammatory cytokines tended to be suppressed (Fig. 4C) and the NF-κB pathway was also suppressed, as evidenced by reduced levels of phosphorylated NF-κB p65 (Fig. 3 and 5), resulting in the reduction of colon shortening and carcinogenesis. As a natural result, *O*-GlcNAcylation was augmented in *Ogt*-Tg mice. It is thought that the inhibition of NF-κB p65 phosphorylation is due to its augmented *O*-GlcNAcylation, and the consequent inactivation of NF-κB p65 leads to the reduction of proinflammatory cytokines. Therefore, the alleviation of colon carcinogenesis by *Ogt* overexpression may be mainly due to augmented *O*-GlcNAcylation.

Various reports have indicated that protein *O*-GlcNAcylation is involved in the tumorigenesis of various tissues. Many molecules that are known to mediate tumorigenesis, including hypoxia-inducible factor-1 (HIF-1), have been implicated in *O*-GlcNAcylation-related tumorigenesis, and reducing OGT and *O*-GlcNAcylation was shown to stabilize HIF-1 in breast cancer cells.^(36)^ *O*-GlcNAcylation enhanced the expression, stability, and function of Yes-associated protein (YAP), the downstream transcriptional regulator of the Hippo pathway and a potent oncogenic factor in liver cancer.^(37)^ Reducing *O*-GlcNAc levels in breast cancer cells also decreased the protein levels of the oncogenic transcription factor FoxM1.^(19)^ In the present study, augmented *O*-GlcNAcylation certainly compromised colon cancer formation in the DMH/DSS-induced inflammatory carcinogenic mouse model. How then does *O*-GlcNAcylation affect carcinogenesis? It is most likely that the effect of *O*-GlcNAcylation varies depending on the tissue. With respect to colon cancer, in which inflammation is clearly involved in carcinogenesis, *O*-GlcNAcylation may downregulate the carcinogenesis pathways and upregulate tumor growth. In other words, when *O*-GlcNAcylation is augmented by *Ogt* overexpression, the incidence of cancer development is decreased; however, once cancer does develop, it may grow relatively quickly.

Protein *O*-GlcNAcylation has also been strongly implicated in regulating inflammation, either negatively or positively. The *O*-GlcNAcylation of NF-κB has been intensively studied,^(38,39)^ and has been shown to accelerate inflammation in some cases,^(22)^ but to not affect or even suppress inflammation in other cases.^(23,24)^ It is considered that the *O*-GlcNAcylation of NF-κB mechanically inhibits its phosphorylation.^(11)^

The role of NF-κB *O*-GlcNAcylation in inflammation is also controversial in colon carcinogenesis models. It was recently reported that inflammatory carcinogenesis was enhanced in heterozygous *Oga* knockout mice, where protein *O*-GlcNAcylation shows a higher basal level, as well as in *Ogt*-Tg mice.^(40)^ In contrast, our results showed that DMH/DSS-induced colon carcinogenesis was suppressed in *Ogt*-Tg mice, where protein *O*-GlcNAcylation was elevated. It is not easy to explain these discrepant results. One possible reason is that the pattern of enhanced protein *O*-GlcNAcylation induced by the *Ogt* transgene and *Oga* suppression might show some qualitative differences, such as different targets or turnover rates. Given that OGT catalyzes the site-specific proteolysis of HCF-1,^(41)^ which regulates the cell cycle, another function of OGT might produce the opposite result. Another possibility is that the intensity of inflammation in the induced colon carcinogenic mouse models used in the different studies might be different. Further studies are necessary to resolve this controversy.

In experiments using the DMH/DSS-induced colon carcinogenic model, the expression levels of proinflammatory cytokines such as *Il-1β*, *Il-6* and *Tnf-α* were not significantly changed. There are several potential reasons for this unexpected result. First, it was very difficult to reach statistical significance because the mRNA expression levels showed wide individual variation. Second, the inflammation in the colonic tissues settled down by 28 days after the second period of DDS treatment. Third, the colonic mucosa was severely shedding. Therefore, a large part of the colonic mucosa, which is considered to cause severe inflammation, had come off.

In conclusion, we revealed that enhanced *Ogt* expression could suppress inflammatory carcinogenesis in the colon through inhibition of the NF-κB pathway, implicating that OGT overexpression and the subsequent increased protein *O*-GlcNAcylation could be new therapeutic targets for colon cancer. Given that augmented *O*-GlcNAcylation is also known as a potent accelerator of cancer cells, new *O*-GlcNAcylation-targeting drugs should be developed carefully.

## Author Contributions

Research design: TN, MA, YH, TI, KH, conducted experiments: YH, TN, TI, EK, KK, TT, Performed data analysis: TN, MA, YH, KM, KK, wrote or contributed to the writing of the manuscript: MA, TN, YH, study supervision: KH, MA.

## Figures and Tables

**Table 1:** Sequences of primers used in real-time PCR

Gene name	Forward, 5'-3'	Reverse, 5'-3'
*Il-1β*	CACCTCTCAAGCAGAGCACAG	GGGTTCCATGGTGAAGTCAAC
*Il-6*	TCCTACCCCAACTTCCAATGCTC	TTGGATGGTCTTGGTCCTTAGCC
*Tnf-α*	AAATGGGCTCCCTCTCATCAGTTC	TCTGCTTGGTGGTTTGCTACGAC
*Gapdh*	GTATTGGGCGCCTGGTCACC	CGCTCCTGGAAGATGGTGATGG

## Abbreviations

DMH: dimethyl hydrazine
DSS: dextran sulfate sodium
*O*-GlcNAc: *O*-linked *N*-acetylglucosamine
OGA: β-*N*-acetylhexosaminidase
OGT: *O*-GlcNAc transferase
Tg: transgenic
